# Cellular metabolism therapy

**DOI:** 10.1186/s12967-022-03514-4

**Published:** 2022-07-05

**Authors:** Salvatore Nesci

**Affiliations:** Department of Veterinary Medical Sciences, University of Bologna, Ozzano Emilia, Italy

We are delighted to announce the launch of *Cellular Metabolism Therapy*, a new section of the Journal of Translational Medicine. “From benchside to bedside to community” are the cornerstones of translational medicine to improve the prevention, diagnosis and therapies with the final goal to maintain and improve human health. *Cellular Metabolism Therapy* as a new section aims at promoting the translation of basic research emerging from biochemistry, molecular medicine, physiology and biotechnology into cell metabolism in order to be exploited in clinical applications. Studies considered for publication cover any aspect of translational metabolic research, including (but not limited to): genetic disorders and epigenetic diseases; metabolic derangements causing neurodegeneration; immunometabolism; stroke; cardiovascular diseases; tumours; stem cell bioenergetics; ageing; carbohydrate, lipid, and protein metabolism.

To this end, cells require multiple steps to accomplish molecular transformation. Biochemical reactions involving enzymes in the metabolic pathways allow cells to respond to changing environmental demands and regulate their metabolism. Therefore, metabolism is emerging as a central influencer of multiple disease states in humans. The energy transduction mechanism responsible for cell homeostasis associated with cellular functions takes place in mitochondria and peroxisomes, which are the central metabolic organelles whose decreased function gives rise to severe mitochondrial and peroxisomal diseases [1, 2]. Among these, the features of heterogeneous genetic conditions rely on pathogenic mutations in either mitochondrial or nuclear genomes triggering mitochondrial disorders from defective oxidative phosphorylation [3, 4]. Accordingly, mitochondrial diseases are featured by clinical, biochemical, and genetic heterogeneity. On the contrary, peroxisomes affect major diseases such as neurodegeneration, diabetes, and cancer. However, several diseases that occur in impaired peroxisomes have not been investigated in patients carrying overt peroxisomal dysfunction. The main concerns arise from the small dimensions of peroxisomes and the high interconnection with other organelles with which they also share many central enzymatic activities (e.g., mitochondria and cytosol) making it difficult to dissect the peroxisomal involvement and activity [5]. Consequently, the biochemical characterization in disease states is flawed.

Neurodegeneration is often accompanied by metabolic dysfunction linked to the mechanism of ageing-related disorders stemming from a defective mitochondrial ATP production [6]. Neuronal networks use energy from ATP hydrolysis and brain glucose hypometabolism conduces to neuronal death. In neurodegenerative diseases, a substantial remodelling of the biological capacity of brain cells causes abnormalities in the well-balanced mechanisms, which are harmed by dysmetabolic events. As a result, drug design to target mitochondria represents the new trend in molecular pharmacology for the treatment of neurodegenerative diseases associated with metabolic disorders [7].

Stem cell behaviours (self-renewal, maintenance, proliferation, cell fate determination, and differentiation) are delicately interplayed with the mitochondrial dynamics. As a consequence, stem cell niche factors and stress signalling regulate the responsiveness of mitochondrial dynamics and the anaerobic metabolism (glycolysis) by emphasizing the metabolic control of stemness and differentiation. Research in this field might shed light on potential new applications in stem cell-based therapy [8].

Infectious diseases are counteracted by the humoral immune response. The development and functions of immune cells rely on immunometabolism to rule immunoregulation. However, immune system metabolism of immune cells can also affect organs and regulate whole-body metabolism [9]. In particular, B cells undergo metabolic rewiring in diseases directly or indirectly affecting cell functions. Metabolic reprogramming in immune cells might play an important role in the pathogenesis of various diseases [10]. Metabolites and metabolic networks continually perform multitasking activities in immunometabolism and metabolites can also function as signalling molecules [11]. Therefore, the immune cell response is dependent on the metabolic products obtained from specific intracellular metabolic pathways (*i.e.*, glycolysis, tricarboxylic acid cycle, pentose phosphate pathway, fatty acid oxidation, fatty acid synthesis and amino acid metabolism) [12].

In ischaemic brain damage, stroke, ischemia-reperfusion injury and heart failure, cells undergo a significant modification of oxidative metabolism that induce cell death. Therapies focused on the emerging roles of metabolites that can block metabolic pathways raise the possibility of innovative cerebro-cardiovascular therapies. The discovery of succinate accumulation during ischaemia and then its oxidation upon reperfusion has pointed out the produced mitochondrial superoxide as the main responsible for ischaemia-reperfusion injury. Indeed, the inhibition of reactive oxygen species formation in mitochondria, by blocking the reverse electron transfer during cell respiration, prevents the succinate accumulation during ischaemia and protects against tissue damage upon reperfusion [13].

On balance, an improved understanding of the contribution of cellular metabolism in human diseases constitutes the required basis to develop new therapies to prevent and counteract severe pathologies and provide efficient tools to improve and maintain health and life quality. Therefore, *Cellular Metabolism Therapy* section will be focused on cutting-edge research in cellular homeostasis under normal and pathological conditions to provide tools and clues for the treatment of disorders related to altered metabolic pathways.

Open access, high-level peer-review process and rapid publication are the missions of the Journal of Translational Medicine, thus giving the chance for highly interactive and timely discussion among researchers. *Cellular Metabolism Therapy* section is proud that its editorial board is made up of scientists with expertise in the biomedical research field. The editorial board composition and skills will surely ensure that every published manuscript is of the highest quality and internationally competitive. On behalf of the Editorial Board, I look forward to receiving your interesting contributions to *Cellular Metabolism Therapy* section.

Salvatore Nesci, PhD. Section Editor, Cellular Metabolism Therapy.
